# Addition of Organic Acids to Base Wines: Impacts on the Technological Characteristics and the Foam Quality of Sparkling Wines

**DOI:** 10.3390/molecules28217423

**Published:** 2023-11-03

**Authors:** Paola Domizio, Alessandra Luciano, Antigone Marino, Luigi Picariello, Martino Forino, Francesco Errichiello, Giuseppe Blaiotta, Luigi Moio, Angelita Gambuti

**Affiliations:** 1 Department of Agriculture, Food, Environment and Forestry (DAGRI), University of Florence, Via Donizetti 6, 50144 Firenze, Italy; paola.domizio@unifi.it; 2 Department of Agricultural Sciences, Section of Vine and Wine Sciences, University of Napoli “Federico II”, Viale Italia, 83100 Avellino, Italyforino@unina.it (M.F.); francesco.errichiello@unina.it (F.E.); blaiotta@unina.it (G.B.);; 3 CNR-ISASI and Physics Department, University of Naples Federico II, Via Cinthia Monte S. Angelo, 80126 Naples, Italy; antigone.marino@isasi.cnr.it

**Keywords:** sparkling wines, foam height, foam persistence, organic acid, buffering capacity, tartaric acid, malic acid, lactic acid, citric acid

## Abstract

Climate change is causing a significant decrease in the total acidity of grapes and related wines. This represents a serious issue for sparkling wine production. Consequently, before the second fermentation, the acidification of base wines is often necessary. However, the impacts of the most important organic acids on the foam properties of sparkling wines are not yet well known. The impacts of the addition of tartaric, malic, citric, and lactic acid on the quality of Falanghina and Bombino sparkling wines were evaluated. Analyses were performed soon after the second fermentation and one year after aging *sur lees.* The addition of each different organic acid to the two base wines resulted in significant changes in the sparkling wines not only in terms of pH, titratable acidity, and buffering capacity but also in the content of total amino acids and, in some cases, in the height of the foam and its stability over time. For both grape varieties, acidified wines showed a lower content of total amino acids in comparison with the control wines. The addition of lactic acid determined a higher persistency of the foam even after one year of aging *sur lees* only in Falanghina wines. The results obtained herein highlight the importance of organic acids and the pH of the base wines for the content of amino acids in sparkling wines. No strict correlation between organic acid addition and the foamability of wines was observed.

## 1. Introduction

The increasing interest of consumers in sparkling wines has enhanced the global market’s demand. In detail, in 2018, sparkling wine production reached 20 thousand hectoliters, with an overall worldwide growth of 57% compared with 2002. Almost half of the total volume produced in 2018 came from Italy (27%) and France (22%) [1].

The traditional method for sparkling wine production consists of a second fermentation process of a base wine. This base wine is bottled along with a solution containing sugars and yeasts (*liqueur de tirage*) and left to undergo a second alcoholic fermentation inside the sealed bottle under anaerobic conditions, followed by aging *sur lees* for several months [2].

The production of sparkling wines, as well as other oenological products, has been strongly affected by climate change in recent decades, causing the acceleration of the phenological phases of grape ripening. This has an impact on the compositional characteristics of the grape juice and on the qualitative characteristics of the final product, with an increase in both the sugar concentration and pH and a decrease in titratable acidity. All these changes negatively affect the quality of sparkling wines. Indeed, in comparison with still wines, base wines suitable for producing quality sparkling wines must necessarily have high acidity and low pH values in order to meet consumers’ preference for wine “freshness” [3] and to guarantee the correct evolution during aging. This is the case for some sparkling wines obtained from grape varieties from Southern Italy such as Falanghina, which, in past decades, has shown higher values of titratable acidity compared with current years [4]. Moreover, scientific evidence has reported the impact of organic acids on the foaming ability of sparkling wines [5]. For instance, Pueyo et al. [6] found that tartaric acid is positively correlated with the foam height. On the other hand, the visual properties of sparkling wines, such as the foam height and the foam persistence [7], represent the first attributes observed by consumers.

In this scenario, it is necessary to evaluate the effect of the acidification practice. Although acidification is regulated by the *International Organization de la Vigne et du Vin (OIV)*, little is currently known about the effect of organic acid addition on the chemical composition and foam properties of sparkling wines. To the best of our knowledge, no systematic comparison between wines with different organic acids added has been carried out. The aim of the present work was to evaluate the evolution over time of the main analytical parameters and the foam of sparkling wines in terms of persistence and height. The effect of the addition of organic acids was evaluated soon after the end of the second fermentation and, again, after one year of aging *sur lees*. The experimental trials were carried out by separately adding different organic acids, including tartaric, malic, citric, and lactic acid, into two different base wines obtained from Bombino and Falanghina grapes, typical varieties from Southern Italy.

## 2. Results and Discussion

### 2.1. Analytical Parameters and Buffering Capacity

**Figure 1:**
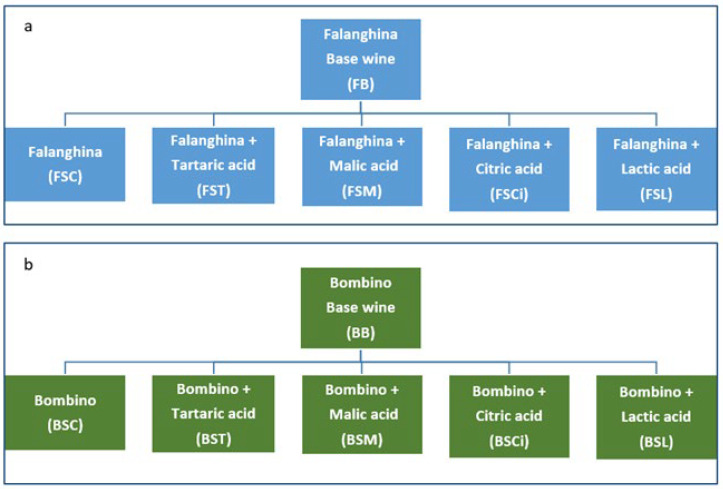
Scheme of the experimental trials for Bombino (**a**) and Falanghina (**b**) grape varieties.

For each variety, a wine without acid addition was used as a reference (control) (Figure 1). Each experimental wine was produced in duplicate.

Table 1 shows the main analytical parameters of the two base wines. The Falanghina base wine (FB) showed higher values of pH and total acidity compared with the Bombino base wine (BB). As expected, the addition of the organic acids determined in all wines an increase in titratable acidity (Table 2). However, the acidification of the Bombino base wine (BB) resulted in a lower pH decrease than that observed in the acidified Falanghina wine (FB), resulting in a pH decrease of approximately 0.1 unit.

**Figure 2:**
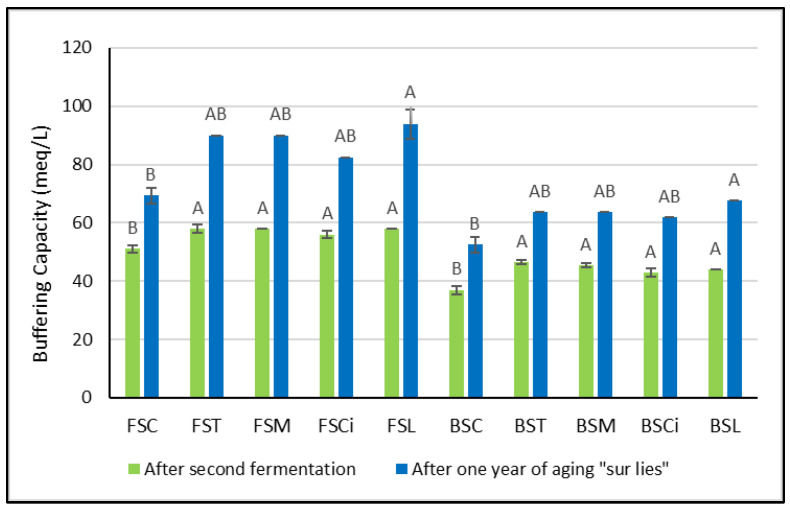
Buffering capacity soon after the organic acid additions and one year after the second fermentation. Significant differences (*p* < 0.05) in buffering capacity in each group of monovarietal wines are expressed with a capital letter (A, B), and the effect of one year of aging *sur lees* was significant for each experimental wine. FSC (Falanghina Control), FST (Falanghina added of tartaric acid), FSM (Falanghina added of Malic acid), FSCi (Falanghina added of Citric acid), FSL (Falanghina added of Lactic acid), BSC (Bombino Control), BST (Bombino added of tartaric acid), BSM (Bombino added of Malic acid), BSCi (Bombino added of Citric acid) and BSL (Bombino added of Lactic acid).

Soon after the organic acid additions, greater variations in buffering capacity were observed in Bombino wines with respect to the control wines (Figure 2). However, no significant differences between the acidified wines were detected. After one year of aging *sur lees*, significant variations in buffering capacity with respect to the control wines were observed only in Falanghina and Bombino wines acidified with lactic acid (FSL and BSL). It is worth mentioning here that Obreque-Slier et al. [8] showed that the buffering capacity of wine prevails over that of saliva, and the pH of the wine/saliva mixture during degustation corresponds to that of the respective wine. Thus, the higher the buffering capacity of a wine, the greater the acid perception. Therefore, in wines treated with lactic acid (FSL and BSL), a longer perception of sourness might be expected.

The data also show that soon after the organic acid additions, the wines treated with lactic acid showed lower values of titratable acidity in comparison with all the other acidified wines. These differences might be due to the different dissociation constant of each organic acid added and the salification equilibrium that each specific acid mixture establishes in a solution. However, after one year of aging *sur lees*, no significant differences between all wines in the titratable acidity values were observed. It should be also taken into consideration that the different organic acids added to the base wines may have had different impacts on the metabolism of the *Saccharomyces cerevisiae* yeast strain that led to the second fermentation. Indeed, *S. cerevisiae* has different uptake systems for monocarboxylic acids, such as acetic and lactic acid, while dicarboxylic acids are assumed to enter the cell in an undissociated form since the presence of permease in *Saccharomyces* has not been demonstrated. Therefore, the undissociated acid crosses the cell membrane and then dissociates due to the higher pH of the cytosol, resulting in both cytoplasmic acidification and the intracellular accumulation of the acid anion. In any case, a different impact of the type of organic acid added might be observed soon after the end of the second fermentation because during aging *sur lees*, autolysis results in the leaching of cellular constituents into the wine [9].

After one year of aging *sur lees*, all acidified Falanghina wines showed a titratable acidity decrease, while this decrease was significant only for the BST and BSM wines (Table 2). In general, this decrease is linked to residual yeast enzymatic activity or to the partial salification of the organic acids due to the precipitation of potassium and calcium tartrate salts during aging *sur lees*.

The data on the individual organic acids (Table 3) show significant changes. In particular, the contents of malic and citric acids were lower than expected after the addition of 2 g/L of each acid. This may be due to the ability of *Saccharomyces* yeast strains to degrade malic and citric acid [10]. This observation is also consistent with the lactic acid contents in FSM and BSM, which were similar to those in the corresponding control wines (FSC and BSC), thus excluding any malolactic bacterial contamination.

Regarding the amino acid profiles, when compared with the Bombino wines, in the Falanghina wines, a higher content of amino acids was detected, with the exception of alanine, valine, methionine, and cysteine. In particular, the most abundant amino acid was tyrosine, followed by proline and GABA (γ-aminobutyric acid) (Table 4). Meanwhile, in the Bombino wines, the most abundant amino acid was GABA, followed by proline and valine (Table 5). The abundance of proline in the wines was expected because it is poorly assimilated by yeasts during the anaerobic conditions of fermentation. Meanwhile, the different contents of tyrosine in the two wines may be linked to the grape varieties, ripening degrees, climates, and geographical origins [11]. Wide differences in tyrosine values in white wines have been reported in the scientific literature and are mainly linked to the grape variety [12,13].

Concerning the impact of acidification on the amino acid contents, significant differences were found for eleven amino acids in the Falanghina wines and five amino acids in the Bombino wines (Table 4 and Table 5).

In general, the acidified wines showed lower contents of amino acids in comparison with the control wines. Interestingly, the amounts of GABA, proline, and leucine were present at significantly lower concentrations in most of the acidified wines, regardless of the grape variety. The higher content of GABA in the control wines might be due to its release into the extracellular environment as a consequence of cell lysis. Indeed, *Saccharomyces* yeasts can utilize GABA as a carbon source [14]. Hence, the lower content of GABA in all the acidified wines might indicate that it was accumulated in the intracellular pool, undergoing autolysis more slowly than when present in the non-acidified wines.

However, as already pointed out by Feuillat [15], amino acids are not good markers for following autolysis due to it being difficult to distinguish between the various stages of the release of the amino acids. Therefore, the lower contents of amino acids in the acidified wines compared with the corresponding control wines may also point to the fact that the yeasts in these wines reached an advanced autolysis stage more quickly, with the consequent reactions of deamination and decarboxylation.

Moreover, nitrogen compound release is thought to reflect the autolytic activity of yeast, especially its proteolytic activity. In this context, the yeast enzymatic activity might have also been differently affected by the low pH values [16] of the acidified wines.

Therefore, further investigations are necessary to understand the impact of acidification treatment on yeast autolysis during aging *sur lees.*

### 2.2. Foam Quality

**Figure 3:**
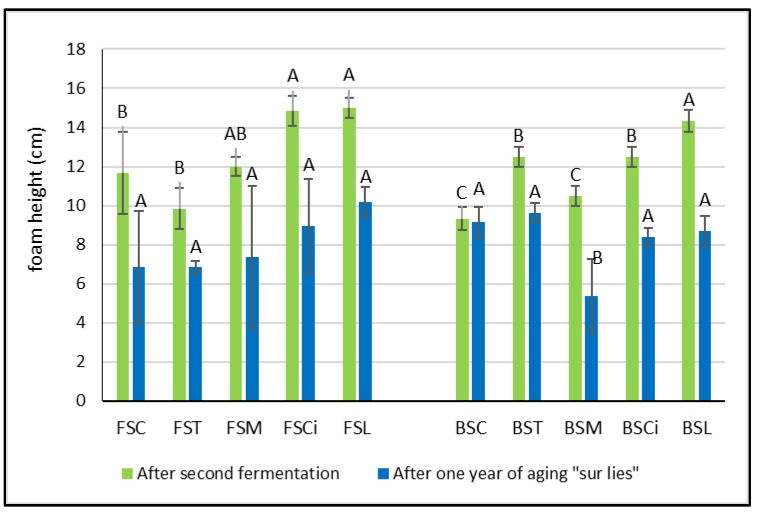
Foam height in experimental wines after the second fermentation and after one year *sur lees*. Significant differences (*p* < 0.05) in buffering capacity in each group of monovarietal wines are expressed with a capital letter (A–C). FSC (Falanghina Control), FST (Falanghina added of tartaric acid), FSM (Falanghina added of Malic acid), FSCi (Falanghina added of Citric acid), FSL (Falanghina added of Lactic acid), BSC (Bombino Control), BST (Bombino added of tartaric acid), BSM (Bombino added of Malic acid), BSCi (Bombino added of Citric acid) and BSL (Bombino added of Lactic acid).

After the end of the second fermentation, the height of the foam varied according to the different types of acid added and to the type of wine. In the Falanghina wines, in comparison with the control wine, the addition of tartaric acid (FST) and malic acid (FSM) determined a decrease in the foam height, even if not significant (Figure 3). Meanwhile, in the Bombino wines, after the second fermentation, the addition of organic acids always determined a significant increase in the foam height, with the exception of BSM (Figure 3). These results related to the impact of malic acid on the height of the foam seem to not be in agreement with those reported by other authors and obtained with other grape varieties [5,17,18,19]. However, in these studies, the relationship between the foam height and malic acid content of wines was related to the base wines that did or did not undergo malolactic fermentation and not to a comparison between the wines with or without added malic acid.

**Figure 4:**
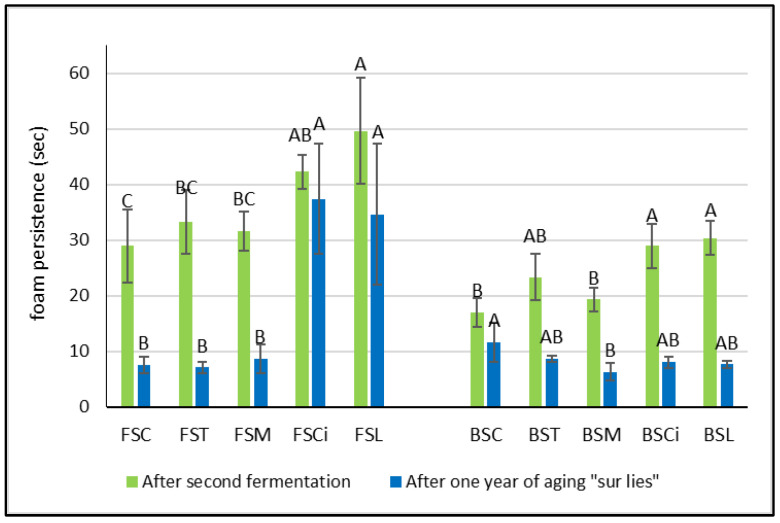
Foam persistence in experimental wines after the second fermentation and after one year *sur lees*. Significant differences (*p* < 0.05) in foam persistency in each group of monovarietal wines are expressed with a capital letter (A–C). FSC (Falanghina Control), FST (Falanghina added of tartaric acid), FSM (Falanghina added of Malic acid), FSCi (Falanghina added of Citric acid), FSL (Falanghina added of Lactic acid), BSC (Bombino Control), BST (Bombino added of tartaric acid), BSM (Bombino added of Malic acid), BSCi (Bombino added of Citric acid) and BSL (Bombino added of Lactic acid).

Interestingly, after the end of the second fermentation, in both the Falanghina and Bombino wines added with citric and lactic acid, the persistence of the foam was significantly higher in comparison with the other acidified wines (Figure 4a). In particular, the Falanghina wine added with lactic acid (FSL) resulted in the sparkling wine with the highest foam persistence, showing approximately a 70% increase in foam persistence compared with that of the control wine. These results are in agreement with those of other authors who found that higher concentrations of lactic acid were positively correlated with foam persistence [17,18,20]. However, after one year of aging *sur lees*, a general decrease in both parameters (foam height and foam persistence) was observed in all wines, and lower differences between the two varieties were registered (Figure 3 and Figure 4). Interestingly, the Falanghina wine with added lactic acid (FSL) showed the highest value of foam persistence compared with all the other wines (Figure 4), similar to what was observed in the wines after the second fermentation. These effects might be linked to the change in viscosity due to the lactic acid addition [21]. Some authors have shown that the viscosity of a bubble’s film is directly related to the wine’s viscosity [22,23], and viscosifying substances provide firmness to the bubbles [24]. Besides organic acids, other factors can also affect the characteristics of the foam. Indeed, tensioactive molecules present in wine, such as ethanol, glycerol, fatty acids, and others, are able to reduce the wine’s surface tension, enabling bubbles to accumulate on the wine’s surface. The hydrophilic groups of these tensioactive molecules dissolve in the aqueous phase of both the wine and the film surrounding the bubble, while the hydrophobic groups that are insoluble in water have to be in the gaseous phase [24]. Also, citric acid, acting as a chelating agent [25], might determine an increase in foamability, as has been observed in beer production [26], probably due to the chelation of metals, with a consequent decrease in surface tension. Moreover, compounds such as proteins, amino acids, and polysaccharides are reported to provide viscosity, and, therefore, elasticity, to the film surrounding a bubble, strengthening its resistance to rupture.

**Figure 5:**
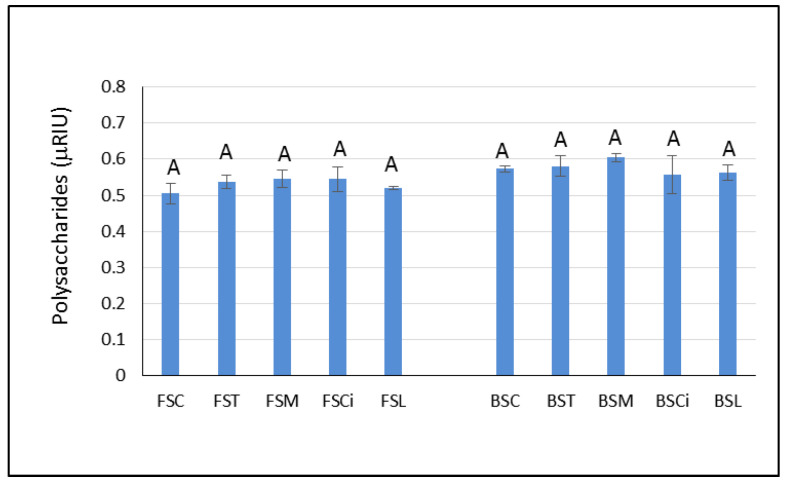
Peak area units of polysaccharides, expressed as refractive index units (RIUs), in the experimental wines after one year of aging *sur lees.* Significant differences (*p* < 0.05) in the areas under the chromatographic peaks associated with total polysaccharides in each group of monovarietal wines are expressed with a capital letter (A). FSC (Falanghina Control), FST (Falanghina added of tartaric acid), FSM (Falanghina added of Malic acid), FSCi (Falanghina added of Citric acid), FSL (Falanghina added of Lactic acid), BSC (Bombino Control), BST (Bombino added of tartaric acid), BSM (Bombino added of Malic acid), BSCi (Bombino added of Citric acid) and BSL (Bombino added of Lactic acid).

Although polysaccharides are reported to be important compounds for foam quality, in the present study, they did not seem to be responsible for the higher persistence of the foam observed in the Falanghina wines with added citric (FSCi) and lactic acid (FSL). Indeed, the area under the chromatographic peak associated with the total polysaccharides content [27] was similar in both wines (Figure 5). However, it is not possible to exclude the impact of polysaccharides on the foamability of the experimental sparkling wines evaluated herein. Indeed, the analysis performed in the present study was not able to highlight differences in the type of polysaccharides that could have been involved in the stability of the foam. Some authors [28,29] found that polysaccharides in the range of 5–100 kDa MW were capable of forming an adsorption layer at the interface with gases, contributing to the stability of the sparkling wine bubbles. Therefore, future research is needed to characterize the molecular weights of these polysaccharides and to evaluate their chemical compositions.

The acidification treatment might have had an impact on the protein charge. Accordingly, some authors [30] have shown that the positive charge on proteins at wine pH levels enables them to migrate to the wine/air interface, stabilizing the foam. Because the protein charge is pH-dependent, the decrease in the wine pH as a consequence of the addition of organic acids might have determined an increase in the positive protein charge. Indeed, the wines acidified with lactic acid (FSL and BSL) and citric acid (FSCi and BSCi) were the wines with the lowest pH values with respect to the related control wines.

Moreover, the greater foam height and persistence detected after the second fermentation in the Falanghina wines compared with the Bombino wines could be linked to the higher amounts of amino acids found in these wines (Table 4 and Table 5). Accordingly, previous studies reported a positive correlation between the total amino acids content and the foam height and foam stability in sparkling wines [31]. Also, the higher content of tyrosine found in the Falanghina wines (Table 4) might be responsible for the foam persistence detected after the second fermentation, in agreement with the results reported by other authors [31]. However, the important role of specific mannoproteins deriving from yeast lysis and grape native proteins [29] cannot be ruled out.

## 3. Materials and Methods

### 3.1. Experimental Wines

The sparkling wines were obtained using the traditional method, as reported below.

#### 3.1.1. Base Wines and Acidification Treatments

Two Italian grape varieties, Bombino and Falanghina, were supplied by “Masseria del Sole”, a winery located in the province of Foggia, Italy. The grapes were processed in the winery according to the internal protocol. After filtration, 2 g/L each of tartaric, malic, citric, and lactic acid was separately added to the two base wines. The addition of 2 g/L of each acid was a compromise between the target pH values ranging from 2.8 to 3.0 and an increase in titratable acidity under the limit of 4 g/L, as defined by the current regulations.

#### 3.1.2. Sparkling Wine Production

All experimental wines (using 10 L stainless tanks for each experimental trial) were supplemented with sucrose (2.4% *w*/*v*). The *Saccharomyces cerevisiae* Lalvin EC1118 strain (Lallemand Inc., Montreal, Canada) was used to start the second fermentation. The yeasts were rehydrated following the manufacturer’s instructions and cultured in wine for 48 h before being added to the wines (*pied de cuve*). A microscopic cell count was carried out to calculate the *pied de cuve* volume needed to obtain a concentration of ~5 × 10^6^ cells/mL of inoculum in the tank. In each tank, 250 mL of *pied de cuve* was added. After that, the wine samples were bottled (0.375 Ls-green glass bottles) and then provisionally capped with bidules and crown caps. Two bottles for each experimental trial were closed with an aphrometer to measure the pressure inside the bottles every two days.

All the samples were stored horizontally at 16 °C and at a relative humidity of 75–85% for 12 months. After one year of aging *sur lees*, the bottoms of the bottles were turned 1/4 clockwise for a month every other day.

### 3.2. Wine Analyses

All analyses were carried out soon after the second fermentation and after one year of aging *sur lees*. Two bottles of each experimental wine were analyzed for each sampling time.

#### 3.2.1. Materials

All solvents were of HPLC grade or higher and were purchased from Merck KGaA, Darmstadt, Germany. The chemicals were of analytical grade (>99%) and were provided by J.T. Baker (Levanchimica, Bari, Italy). All of the amino acid standards and the diethylethoxymethylenemalonate (DEEMM) were purchased from Sigma-Aldrich (Milan, Italy). Water was purified using a Milli-Q purification system (Millipore Sigma, Burlington, MA, USA).

#### 3.2.2. Main Analytical Parameters

The main analytical parameters of the wines were determined as follows: total acidity and volatile acidity according to the official OIV methods; free and total sulfur dioxide and tartaric acid via colorimetric methods using the Y15 BioSystems system (Costa Brava, Barcelona, Spain); and residual sugars, malic acid, citric acid, and d-lactic and l-lactic acid via enzymatic methods using the Y15 BioSystems system (Costa Brava, Barcelona, Spain). The base parameters of the finished wines are shown in Table A1 in Appendix A.

#### 3.2.3. Total Polysaccharides

Total polysaccharides were evaluated via HPLC according to Peyron et al. [27] with some modifications, as previously described [32]: isocratic separation was performed at 65 °C in a Supelco TSK G-OLIGO-PW (808031) column (30 cm × 7.8 mm i.d.) equipped with a Supelco TSKGEL OLIGO (808034) guard column (4 cm × 6 mm i.d.). A mobile phase of 0.2 M sodium chloride at a flow rate of 0.8 mL/min was used. The area under the chromatographic peak was recorded and integrated using Galaxie Chromatography Data System version 1.9.302.530 (Varian Inc., Palo Alto, CA, USA).

#### 3.2.4. Amino Acid Analysis

Standard solutions of each amino acid were prepared in HCl 0.1 N. HPLC-based analyses of amino acids were conducted with an HPLC Agilent 1260 Infinity II LC apparatus (Santa Clara, CA, USA) equipped with a binary pump with an integrated two-channel degasser unit and a diode array detector (DAD) (G7114A). The data collection and analyses were performed using the software OpenLAB CDS ChemStation Edition 3.6 (Agilent Technologies, Santa Clara, CA, USA).

Amino acids were derivatized according to the procedure described by Gomez-Alonso et al. [33] and Ortega-Heras et al. [34]. The HPLC-based identification of amino acids was carried out via comparison with amino acid standards. For the HPLC analyses, an Agilent Infinity Lab Poroshell 120 EC C18 (3.0 × 150 mm, 2.7 μm) was used. Eluent A: 25 mM of acetate buffer (pH 5.8) with 0.02% sodium azide; eluent B: acetonitrile/methanol 8:2 (*v*/*v*). Flow rate: 0.5 mL/min. For detection, λ = 280 nm was used. Eluent B was used with the following gradient: 5% from 0 to 20 min; 10% from 20 to 30 min; 17% from 30 to 33 min; 40% from 33 to 65 min; 72% from 65 to 73 min; 82% from 73 min to 78 min; and, finally, 100% from 82 min to 87 min. After that, eluent B was returned to the starting percentage. Amino acids were identified and quantified on the basis of the retention times and UV spectral properties of the derivatives of the respective standards.

#### 3.2.5. Buffering Capacity

The buffering capacity (π), defined as the resistance of a solution to pH changes following the addition of an acid or an alkali, was calculated as follows:

π = (dB)/(dpH)

π is the amount of base (B) needed to increase the pH of the wine by one unit.

For the calculation of the wine buffering capacity, the consumed mL of NaOH (1N) and the pH changes were considered. The buffering capacity was expressed in meq/L.

#### 3.2.6. Foam Quality

Foam quality was evaluated in terms of height and persistence. New ISO glasses with no faults or marks were used. The glasses were rinsed first with hot water and then with room-temperature distilled water. Finally, they were arranged upside down for drying via water evaporation. Neither paper nor cloth was used in the drying process, nor was a dishwasher used, because alkaline detergents are very hard on glass, and the shine components of detergents could modify the foaming properties, as described by Gallart et al. [35]. The foam height was evaluated via visual analysis performed using an optical bench and two reflex cameras, a Nikon 800 D reflex camera with a 55 mm macro lens, and a Nikon 850 reflex camera with a 50 mm lens. The glasses were illuminated with three LED lamps (850 lumen) with white light (4000 K). The maximum foam height and persistence were evaluated with the Nikon 850 reflex camera, placed in a horizontal position, at 38 cm from the glass and with a sensor sensitivity to light ISO of 1600, with an exposure time of 1/60, and with an aperture of f = 22. In particular, the foam height upon pouring was measured from the base of the collar to its highest point. The foam persistence, that is, the time the bubbles take to entirely collapse and, hence, for the foam to disappear, was measured over time (seconds).

#### 3.2.7. Data Analysis

Statistical analysis was performed using XLSTAT (software Addinsoft, 2017.1). The influence of the treatments was assessed via analysis of the variance (ANOVA) using Tukey’s method for the significant differences in the procedures (*p* < 0.05). All data are means of four values: two experimental replicas and two analytical replicas.

## 4. Conclusions

Musts obtained in temperate to hot climates often need to be acidified. Usually, tartaric and/or citric acid are added. In the present study, important differences were observed following the addition of other types of acids before the second fermentation. In particular, lactic acid, and, to a lesser extent, citric acid, led to greater foaming performances. Besides the impact on the foam, lactic acid also showed the highest buffering capacity. This result is important as it could determine a greater persistence of the acid sensation, an important feature of a sparkling wine.

Based on our results, malolactic fermentation of the base wine before the second fermentation might be favored. In this way, it is possible to obtain not only a natural increase in lactic acid but also the biological stabilization of the wine, preventing it from occurring later in the bottle. After that, further lactic acid addition can be evaluated, exploiting its ability to increase the viscosity of the wine and, therefore, positively impacting the foamability of related sparkling wines.

However, the foam of sparkling wines produced with the traditional method should be evaluated over a long time of aging *sur lees* in order to investigate the evolution over time of the buffering capacity and of the acid sensation persistence.

Finally, the impact of the grape variety on the features of the foam of sparkling wines was also highlighted. Indeed, the Falanghina base wine showed a better foaming performance with respect to the Bombino wines, even if lower differences were observed between the two varieties after one year of aging *sur lees*, probably linked to the different amino acid profiles.

## Figures and Tables

**Table 1:** Main analytical parameters of base wines.

Sample Code	Residual Sugar (g/L)	pH	Ethanol (% *v/v*)	Total Acidity (g/L of Tartaric Acid)	Total SO_2_ (mg/L)
**FSC**	0.26 ± 0.01	3.13 ± 0.01	11.03 ± 0.01	9.82 ± 0.00	49.50 ± 0.71
**BSC**	0.19 ± 0.01	2.98 ± 0.00	11.60 ± 0.01	6.90 ± 0.00	102.00 ± 1.41

FSC (Falanghina Control), BSC (Bombino Control).

**Table 2:** Titratable acidity and pH values of base wines after organic acid addition.

	After Organic Acid Addition		After 1 Year of Aging *Sur Lees*	
Sample Code	pH	Titratable Acidity (g/L of tartaric acid)	pH	Titratable Acidity (g/L of tartaric acid)
**FSC**	3.12 ± 0.02 A	9.79 ± 0.04 C	3.14 ± 0.02 AB	9.90 ± 0.32 B
**FST**	2.96 ± 0.03 B	11.57 ± 0.07 AB	3.26 ± 0.01 A *	10.91 ± 0.05 A *
**FSM**	3.03 ± 0.01 AB	12.06 ± 0.20 A	3.03 ± 0.05 BC	10.99 ± 0.05 A *
**FSCi**	3.02 ± 0.03 B	11.84 ± 0.12 AB	2.90 ± 0.06 C	11.08 ± 0.13 A *
**FSL**	3.03 ± 0.02 AB	11.29 ± 0.04 B	2.98 ± 0.02 C	10.78 ± 0.08 A *
**BSC**	2.98 ± 0.03 A	6.83 ± 0.11 C	2.99 ± 0.04 A	6.84 ± 0.08 C
**BST**	2.88 ± 0.02 B	8.95 ± 0.06 A	2.82 ± 0.03 B	8.44 ± 0.00 AB *
**BSM**	2.94 ± 0.01 AB	9.04 ± 0.04 A	2.91 ± 0.03 AB	8.44 ± 0.05 AB *
**BSCi**	2.90 ± 0.03 AB	9.11 ± 0.06 A	2.81 ± 0.03 B	8.96 ± 0.21 A
**BSL**	2.93 ± 0.02 AB	8.66 ± 0.06 B	2.66 ± 0.01 C	8.31 ± 0.19 B

Significant differences (*p* < 0.05) in pH and titratable acidity in each group of monovarietal wines are expressed with a capital letter (A–C), and the effect of one year of aging *sur lees* is expressed with an asterisk (*). FSC (Falanghina control), FST (Falanghina with added tartaric acid), FSM (Falanghina with added malic acid), FSCi (Falanghina with added citric acid), FSL (Falanghina with added lactic acid), BSC (Bombino control), BST (Bombino with added tartaric acid), BSM (Bombino with added malic acid), BSCi (Bombino with added citric acid), and BSL (Bombino with added lactic acid).

**Table 3:** Contents of organic acids (g/L) in Falanghina and Bombino wines one year after the second fermentation.

Sample Code	Tartaric Acid	Malic Acid	Citric Acid	d-Lactic Acid	l-Lactic Acid
**FSC**	3.08 ± 0.01 b	5.14 ± 0.02 b	0.46 ± 0.48 b	0.09 ± 0.00 b	0.02 ± 0.00 b
**FST**	5.21 ± 0.02 a	5.01 ± 0.08 b	0.47 ± 0.47 b	0.09 ± 0.00 b	0.01 ± 0.00 b
**FSM**	2.97 ± 0.03 c	6.19 ± 0.09 a	0.48 ± 0.48 b	0.09 ± 0.00 b	0.01 ± 0.00 b
**FSCi**	2.99 ± 0.01 c	5.15 ± 0.02 b	1.67 ± 0.02 a	0.09 ± 0.00 b	0.01 ± 0.00 b
**FSL**	2.99 ± 0.01 c	5.13 ± 0.08 b	0.48 ± 0.48 b	0.14 ± 0.00 a	1.75 ± 0.03 a
**BSC**	4.73 ± 0.02 b	1.40 ± 0.03 b	0.26 ± 0.23 b	0.11 ± 0.00 a	0.01 ± 0.00 b
**BST**	6.80 ± 0.02 a	1.41 ± 0.02 b	0.23 ± 0.23 b	0.11 ± 0.00 b	0.01 ± 0.00 b
**BSM**	4.55 ± 0.02 d	3.16 ± 0.06 a	0.24 ± 0.23 b	0.11 ± 0.00 b	0.02 ± 0.01 b
**BSCi**	4.60 ± 0.01 c	1.41 ± 0.02 b	1.58 ± 0.01 a	0.11 ± 0.00 b	0.01 ± 0.00 b
**BSL**	4.58 ± 0.01 cd	1.41 ± 0.04 b	0.24 ± 0.24 b	0.16 ± 0.00 b	1.82 ± 0.08 a

For each group of monovarietal wines, the effect of organic acid addition for the same organic acid content is expressed with a lowercase letter (a–d) (*p* < 0.05). FSC (Falanghina Control), FST (Falanghina added of tartaric acid), FSM (Falanghina added of Malic acid), FSCi (Falanghina added of Citric acid), FSL (Falanghina added of Lactic acid), BSC (Bombino Control), BST (Bombino added of tartaric acid), BSM (Bombino added of Malic acid), BSCi (Bombino added of Citric acid) and BSL (Bombino added of Lactic acid).

**Table 4:** Contents of amino acids (mg/L) in Falanghina wines after one year of aging *sur lees*.

	Control	Tartaric Acid	Malic Acid	Citric Acid	Lactic Acid
**Aspartic acid**	6.56 ± 0.24 a	6.49 ± 0.31 a	5.94 ± 0.23 a	5.83 ± 0.04 a	6.43 ± 0.15 a
**Glutamic acid**	5.74 ± 0.18 a	5.83 ± 0.29 a	5.18 ± 0.27 a	5.32 ± 0.03 a	5.47 ± 0.24 a
**Asparagine**	4.72 ± 0.52 b	6.14 ± 0.31 a	6.56 ± 0.28 a	6.61 ± 0.25 a	5.85 ± 0.07 ab
**Serine**	3.55 ± 0.35 a	3.58 ± 0.15 a	3.29 ± 0.22 a	3.23 ± 0.11 a	3.42 ± 0.22 a
**Glutamine**	2.83 ± 0.33 a	3.35 ± 0.08 a	3.27 ± 0.12 a	2.82 ± 0.34 a	3.08 ± 0.26 a
**Histidine**	8.73 ± 0.38 ab	7.87 ± 0.27 bc	8.97 ± 0.09 a	7.15 ± 0.14 c	7.94 ± 0.21 c
**Glycine**	4.88 ± 0.28 a	4.04 ± 0.26 a	4.30 ± 0.33 a	4.24 ± 0.26 a	4.89 ± 0.26 a
**Threonine**	9.36 ± 1.11 a	9.93 ± 0.45 a	9.12 ± 0.43 a	9.55 ± 0.13 a	10.42 ± 0.06 a
**Arginine**	8.35 ± 0.20 a	8.44 ± 0.31 a	9.26 ± 0.35 a	9.18 ± 0.38 a	9.25 ± 0.29 a
**α-Alanine**	9.58 ± 0.74 a	9.57 ± 0.04 a	9.95 ± 0.37 a	9.78 ± 0.15 a	10.11 ± 0.07 a
**GABA**	35.69 ± 0.69 ab	36.41 ± 0.89 a	32.79 ± 0.34 c	32.44 ± 0.16 c	33.73 ± 0.25 bc
**Proline**	45.16 ± 2.95 a	41.84 ± 0.40 ab	37.55 ± 0.57 b	37.15 ± 0.31 b	41.78 ± 0.30 ab
**Tyrosine**	54.63 ± 1.37 a	53.92 ± 1.23 a	44.65 ± 0.39 b	45.11 ± 0.30 b	38.36 ± 0.46 c
**Valine**	6.98 ± 0.25 a	6.24 ± 0.12 b	6.30 ± 0.14 ab	6.33 ± 0.14 ab	6.98 ± 0.18 a
**Methionine**	6.30 ± 0.12 a	4.80 ± 0.26 b	3.76 ± 0.23 c	4.16 ± 0.18 bc	4.28 ± 0.30 c
**Cysteine**	5.47 ± 0.11 ab	5.76 ± 0.18 ab	5.84 ± 0.05 a	5.82 ± 0.10 ab	5.37 ± 0.11 b
**Isoleucine**	5.80 ± 0.22 a	5.23 ± 0.04 ab	4.06 ± 0.21 c	3.97 ± 0.10 c	4.52 ± 0.28 bc
**Tryptophan**	4.77 ± 0.52 a	4.82 ± 0.33 a	4.46 ± 0.11 a	4.52 ± 0.05 a	4.01 ± 0.10 a
**Leucine**	7.13 ± 0.18 a	7.11 ± 0.23 a	6.42 ± 0.27 ab	6.05 ± 0.23 b	6.18 ± 0.21 b
**Phenylalanine**	5.46 ± 0.27 a	4.90 ± 0.18 a	5.01 ± 0.37 a	5.21 ± 0.25 a	4.68 ± 0.33 a
**Lysine**	10.84 ± 0.31 a	10.04 ± 0.29 ab	9.15 ± 0.22 b	9.09 ± 0.08 b	9.34 ± 0.36 b

Significant differences (*p* < 0.05) between wines for each amino acid are expressed with a lowercase letter (a–c). FSC (Falanghina Control), FST (Falanghina added of tartaric acid), FSM (Falanghina added of Malic acid), FSCi (Falanghina added of Citric acid), FSL (Falanghina added of Lactic acid), BSC (Bombino Control), BST (Bombino added of tartaric acid), BSM (Bombino added of Malic acid), BSCi (Bombino added of Citric acid) and BSL (Bombino added of Lactic acid).

**Table 5:** Contents of amino acids (mg/L) in Bombino wines after one year of aging *sur lees*.

	Control	Tartaric Acid	Malic Acid	Citric Acid	Lactic Acid
**Aspartic acid**	4.42 ± 0.43 a	3.74 ± 0.52 a	3.76 ± 0.06 a	4.19 ± 0.15 a	4.12 ± 0.35 a
**Glutamic acid**	3.23 ± 0.52 a	2.40 ± 0.48 a	2.45 ± 0.15 a	2.13 ± 0.15 a	2.90 ± 0.02 a
**Asparagine**	3.47 ± 0.25 a	3.42 ± 0.24 a	3.55 ± 0.05 a	3.49 ± 0.19 a	3.61 ± 0.04 a
**Serine**	1.56 ± 0.02 b	1.95 ± 0.12 a	2.16 ± 0.06 a	2.10 ± 0.10 a	2.16 ± 0.13 a
**Glutamine**	0.66 ± 0.06 a	0.70 ± 0.08 a	0.84 ± 0.05 a	0.82 ± 0.06 a	0.69 ± 0.05 a
**Histidine**	2.57 ± 0.32 a	2.58 ± 0.27 a	2.48 ± 0.17 a	2.47 ± 0.15 a	2.68 ± 0.02 a
**Glycine**	3.22 ± 0.32 a	2.99 ± 0.25 ab	2.60 ± 0.02 ab	2.30 ± 0.24 b	2.63 ± 0.02 ab
**Threonine**	1.21 ± 0.18 a	1.28 ± 0.02 a	1.49 ± 0.04 a	1.22 ± 0.17 a	1.25 ± 0.16 a
**Arginine**	3.91 ± 0.50 a	4.00 ± 0.99 a	3.73 ± 0.07 a	3.64 ± 0.06 a	4.14 ± 0.09 a
**α-alanine**	9.50 ± 0.86 a	9.80 ± 0.53 a	8.23 ± 0.20 a	8.56 ± 0.30 a	9.14 ± 0.02 a
**GABA**	38.83 ± 0.60 a	36.10 ± 0.52 b	39.06 ± 0.53 a	37.80 ± 0.74 ab	38.08 ± 0.08 ab
**Proline**	28.08 ± 0.88 a	21.12 ± 0.11 b	17.24 ± 1.34 b	18.37 ± 0.53 b	19.62 ± 3.04 b
**Tyrosine**	8.85 ± 0.69 a	8.10 ± 0.65 a	8.14 ± 0.86 a	7.64 ± 0.29 a	8.64 ± 0.71 a
**Valine**	25.79 ± 1.64 a	23.62 ± 0.75 a	24.34 ± 0.28 a	25.15 ± 0.46 a	25.73 ± 0.83 a
**Methionine**	12.40 ± 0.40 a	11.88 ± 0.25 a	12.32 ± 1.38 a	11.19 ± 0.08 a	10.87 ± 0.30 a
**Cysteine**	0.95 ± 0.05 a	0.82 ± 0.00 a	0.82 ± 0.05 a	0.72 ± 0.16 a	0.76 ± 0.06 a
**Isoleucine**	1.14 ± 0.20 a	1.26 ± 0.05 a	1.09 ± 0.07 a	0.96 ± 0.15 a	1.20 ± 0.12 a
**Tryptophan**	3.61 ± 0.26 a	2.99 ± 0.21 a	3.05 ± 0.11 a	3.03 ± 0.15 a	3.39 ± 0.02 a
**Leucine**	3.21 ± 0.19 a	2.82 ± 0.11 ab	2.62 ± 0.06 b	2.68 ± 0.10 b	2.96 ± 0.04 ab
**Phenylalanine**	2.21 ± 0.27 a	2.17 ± 0.38 a	1.94 ± 0.04 a	1.85 ± 0.08 a	2.18 ± 0.06 a
**Lysine**	3.00 ± 0.24 a	2.63 ± 0.04 a	2.87 ± 0.33 a	2.58 ± 0.02 a	2.94 ± 0.22 a

Significant differences (*p* < 0.05) between wines for each amino acid are expressed with a lowercase letter (a, b). FSC (Falanghina Control), FST (Falanghina added of tartaric acid), FSM (Falanghina added of Malic acid), FSCi (Falanghina added of Citric acid), FSL (Falanghina added of Lactic acid), BSC (Bombino Control), BST (Bombino added of tartaric acid), BSM (Bombino added of Malic acid), BSCi (Bombino added of Citric acid) and BSL (Bombino added of Lactic acid).

## Data Availability

The data presented in this study are available upon request from the corresponding author.

## Appendix A

**Table A1:** Main analytical parameters of base wines after 1 year of aging *sur lees*.

Sample Code	Residual Sugars (g/L)	Ethanol (% *v*/*v*)	Volatile Acidity (g/L of Acetic Acid)	Total SO_2_ (mg/L)
FSC	1.23 ± 0.04	12.14 ± 0.05	0.17 ± 0.00	33.00 ± 0.00
FST	1.28 ± 0.38	11.66 ± 0.54	0.19 ± 0.01	34.50 ± 0.58
FSM	2.24 ± 0.89	11.03 ± 0.14	0.18 ± 0.00	34.75 ± 2.06
FSCi	1.67 ± 0.30	11.80 ± 0.16	0.32 ± 0.06	33.00 ± 0.82
FSL	1.87 ± 0.05	11.65 ± 0.08	0.34 ± 0.01	34.00 ± 0.00
BSC	3.56 ± 0.17	12.44 ± 0.23	0.33 ± 0.04	85.75 ± 0.96
BST	5.17 ± 0.10	12.49 ± 0.05	0.38 ± 0.01	85.50 ± 0.58
BSM	4.11 ± 0.10	12.56 ± 0.19	0.38 ± 0.00	83.00 ± 1.15
BSCi	3.56 ± 0.34	11.31 ± 0.36	0.35 ± 0.02	86.50 ± 2.38
BSL	3.93 ± 0.66	12.58 ± 0.49	0.47 ± 0.00	75.50 ± 0.58
